# Severe herpesvirus infection beats adult T-cell leukemia/lymphoma

**DOI:** 10.18632/genesandcancer.228

**Published:** 2023-01-19

**Authors:** Tatsuro Jo

**Keywords:** adult T-cell leukemia/lymphoma, HTLV-1, Tax, varicella zoster virus vaccination

Aggressive type adult T-cell leukemia/lymphoma (ATLL) caused by human T-cell lymphotropic virus type 1 (HTLV-1) infection is associated with dismal survival, even after the approval of mogamulizumab (a monoclonal antibody for C-C chemokine receptor 4 antigen). A large number of genetic and epigenetic abnormalities as the results of the pleotropic effects of HTLV-1 Tax and HBZ reportedly are present in ATLL patients [1, 2], which may be the cause of their poor response to intensive chemotherapies. The HTLV-1 sequence is completely distinct from the human genome, making the *HTLV-1* gene products highly immunogenic to humans and targets for the humoral and cellular immunity. Actually, antibodies against the *HTLV-1 gag* and *env* gene products are ubiquitously detected in HTLV-1 carriers. Furthermore, cytotoxic T lymphocytes (CTLs) against HTLV-1 Tax (TaxCTLs) are often observed in long-term survivors with ATLL treated with or without allogeneic hematopoietic stem cell transplantation. Thus, the activation of antitumor cellular immunity may play an important role among long-term survivors with ATLL, an intractable disease.

In patients with ATLL, the CD4/CD8 ratio in T lymphocytes is often inverted due to a decrease in CD4-positive T lymphocytes, including regulatory T lymphocytes (Treg), after treatment with mogamulizumab. Conversely, we have noticed that the CD4/CD8 ratio is almost always inverted in long-term surviving ATLL patients in the pre-Mogamulizumab era. Furthermore, several of these patients developed herpesvirus infections, such as herpes zoster and herpes encephalitis, while their disease was controlled by chemotherapy and subsequently exhibited CD4/CD8 ratio reversal [3]. Herpesvirus infection has been reported to strongly activate host cellular immunity [4, 5]. Therefore, the activation of CD8-positve T lymphocytes including TaxCTLs in these patients may have occurred as a result of generalized cellular immune activation due to herpesvirus infections. TaxCTLs remained in these patients several years after the completion of chemotherapy. The simultaneous identification of the HTLV-1 virus itself suggests that TaxCTLs may also prevent ATLL relapse in these long-term survivors. To translate this phenomenon into actual ATLL practice, we investigated whether TaxCTLs are activated by attenuated varicella zoster virus (VZV) vaccination in ATLL patients (a clinical trial to inspect whether varicella zoster vaccine inoculation induces antitumor immunity in patients with adult T-cell leukemia/lymphoma [VAT study]) [6]. Three indolent type and three aggressive type ATLL patients were registered, with one indolent type and three aggressive type ATLL patients showing activation of TaxCTLs. Although it is not possible to intentionally induce a herpesvirus infection during the ATLL treatment when the disease is under control, planned attenuated VZV vaccination during treatment is feasible. If the attenuated VZV vaccination activates generalized cellular immunity in ATLL patients during or immediately after immunochemotherapy and simultaneously activates anti-ATLL tumor immunity, this could lead to improved treatment outcomes in ATLL.

Tax reportedly plays an important role in the process of ATLL development, but after ATLL development, Tax expression is not necessary for ATLL cell survival, and ATLL cells in some ATLL patients do not express Tax [7]. However, the expression of Tax is reportedly only identifiable by RT-PCR in the quiescent state, but it is sufficiently expressed in the actively proliferating state [8]. Furthermore, TaxCTLs reportedly recognize human leukocyte antigen (HLA)/Tax peptide complexes and exert cell-killing effects, even if their expression levels can only be identified by RT-PCR [9]. Thus, TaxCTLs activated by a herpesvirus infection or attenuated VZV vaccination could have a cell-killing effect on ATLL cells *in vivo*. Even if the ATLL cells do not express Tax, it is possible that the large number of genetic alterations caused by Tax and HBZ could possibly lead to the expression of tumor antigens other than Tax and be captured by the host cellular immunity.

There have been attempts to treat patients by using Tax peptide-pulsed dendritic cell vaccine and adoptive immunotherapy by the Tax-specific *TCR* gene transferred T lymphocytes, and promising results have been reported for each [10, 11]. However, these methods require complicated procedures and cannot be applied to all ATLL patients because they are HLA-restricted. Conversely, our attenuated VZV vaccination method is simple, not HLA-restricted, and can be applied to all ATLL patients (Table 1). Furthermore, an effective treatment for indolent type ATLL is currently lacking, and most patients are followed up without any treatment. However, the findings of the VAT study suggest that treatment with mogamulizumab to reduce Treg followed by attenuated VZV vaccination may activate the cellular immunity responsive to tumor antigens, such as Tax, and may be an effective treatment, even in indolent type ATLL.

**Table 1:** Comparison of the characteristics between ATLL therapies using cellular immunity

	Tax peptide-pulsed dendritic cell vaccine therapy Tax-specific *TCR* gene transferred T lymphocytes therapy	Attenuated VZV vaccination method
Subject	Limited patients (HLA-restricted)	All patients (HLA-non-restricted)
Tumor antigens	Tax peptides	Multiple tumor antigens expressed on ATLL cells including Tax peptides
Procedure	Relatively complicated	Simple

Abbreviations: ATLL: adult T-cell leukemia/lymphoma; TCR: T-cell receptor; VZV: varicella zoster virus; HLA: human leukocyte antigen.

In the VAT study, for safety reasons, attenuated VZV vaccination was performed after the confirmation of the presence of anti-VZV antibodies and adequate cellular immunity as confirmed by the lymphocyte blastoid transformation test, and no adverse reaction was observed. These results may serve as a guide when considering attenuated VZV vaccination in patients with all cancer types.

The HTLV-1 infection is recognized by the host immune system, which produces antibodies to *gag* and *env* gene products, but these antibodies cannot eliminate HTLV-1-infected cells. TaxCTLs are also produced, but HTLV-1-infected cells escape the surveillance of TaxCTLs by controlling Tax expression and accumulate genetic abnormalities over time, eventually becoming ATLL cells. Although contracting herpes simplex or herpes zoster is unpleasant, the mechanism by which these herpesvirus infections can produce a therapeutic effect on refractory ATLL via the activation of the host’s cellular immunity is extremely interesting and worth further study.
